# Pathways from maternal depressive symptoms to adolescent depressive symptoms: the unique contribution of irritability symptoms

**DOI:** 10.1111/jcpp.12395

**Published:** 2015-02-09

**Authors:** Yvonne M. Whelan, Ellen Leibenluft, Argyris Stringaris, Edward D. Barker

**Affiliations:** ^1^ Department of Psychological SciencesBirkbeckUniversity of LondonLondonUK; ^2^ Section on Bipolar Spectrum DisordersDivision of Intramural Research ProgramsNational Institute of Mental HealthNational Institutes of HealthBethesdaMDUSA; ^3^ Department of Child and Adolescent PsychiatryInstitute of PsychiatryPsychology and NeuroscienceKing's College LondonLondonUK; ^4^ Department of PsychologyInstitute of PsychiatryPsychology and NeuroscienceKing's College LondonLondonUK

**Keywords:** Psychopathology, oppositional defiant disorder, depression, development, mother–child relationships

## Abstract

**Background:**

The authors tested three possible pathways linking prenatal maternal depressive symptoms to adolescent depressive symptoms. These pathways went through childhood *Irritability Symptoms*,* Anxiety/Depressive Symptoms* or *Conduct Problems*.

**Method:**

Data were collected from 3,963 mother–child pairs participating in the Avon Longitudinal Study of Parents and Children. Measures include maternal depressive symptoms (pre‐ and postnatal); toddler temperament (2 years); childhood (7–13 years) irritability symptoms, anxiety/depressive symptoms, conduct problems, and adolescent depressive symptoms (16 years).

**Results:**

*Irritability Symptoms*: This pathway linked sequentially – prenatal maternal depressive symptoms, toddler temperament (high perceived intensity and low perceived adaptability), childhood irritability symptoms, and adolescent depressive symptoms. *Anxiety/Depressive symptoms*: This pathway linked sequentially – prenatal maternal depressive symptoms, toddler temperament (negative perceived mood), childhood anxiety/depressive symptoms, and adolescent depressive symptoms. Childhood conduct problems were not associated with adolescent depressive symptoms, above and beyond irritability symptoms and anxiety/depressive symptoms.

**Conclusions:**

Results suggest evidence for two distinct developmental pathways to adolescent depressive symptoms that involve specific early and midchildhood features.

## Introduction

Depression contributes significantly to the global burden of disease and affects people in all communities with an onset that typically occurs in adolescence (Andrade et al., 2003; Kessler et al., 2005; Patel, Flisher, Hetrick, & McGorry, 2007). Indeed, those with adolescent‐onset depression often go on to have recurrent episodes and significant impairment (e.g. Hammen, Brennan, and Keenan‐Miller, 2008). As a result, research has sought to identify early family risk factors and child characteristics that can predict adolescent depressive symptoms, to enable early identification and mobilize preventative intervention measures that focus on early risk factors (e.g. Garber, 2006).

In this research, we tested for three distinct pathways defined by correlated but distinct child characteristics, linking a common family risk factor — prenatal maternal depressive symptoms — to an equifinal outcome of adolescent depressive symptoms. The first pathway that we tested goes through childhood *Irritability Symptoms*; the second, through childhood *Anxiety/Depressive symptoms*; and the third through childhood *Conduct Problems*.

Oppositional defiance in youth is a highly prevalent psychiatric condition with strong associations with a wide range of adult psychiatric illness, including both emotional (e.g. depression) and externalizing disorders (e.g. conduct disorder, and callous‐unemotional traits) (Angold, Costello, & Erkanli, 1999; Loeber, Green, Keenan, & Lahey, 1995; Maughan, Rowe, Messer, Goodman, & Meltzer, 2004). Partly due to the fact that Oppositional Defiant Disorder (ODD) predicts to such a wide range of adjustment difficulties in children, the DSM 5 (American Psychiatric Association, 2013) has suggested a distinction among irritable, headstrong, and hurtful ODD dimensions, as these dimensions appear to associate with distinct outcomes. Importantly, studies suggest that the ODD subdimension of irritability (i.e. has temper outbursts; touchy or easily annoyed; angry or resentful) predicts adolescent and young adult depressive symptoms (Leibenluft, Cohen, Gorrindo, Brook, & Pine, 2006; Leibenluft & Stoddard, 2013; Stringaris & Goodman, 2009a; Whelan, Stringaris, Maughan, & Barker, 2013). In addition, previous studies show predictive associations between adolescent depressive symptoms and other child characteristics such as anxiety/depressive symptoms (e.g. has many worries or often seems worried; often unhappy, depressed or tearful) and conduct problems (e.g. often fights with other children or bullies them, often lies or cheats) (Barker, Oliver, & Maughan, 2010; Goodman, 2001; Lahey, Loeber, Burke, & Rathouz, 2002; Stringaris, Lewis, & Maughan, 2014). Of importance, irritability symptoms are associated with child anxiety/depressive symptoms and conduct problems (Dougherty et al., 2013; Krieger et al., 2013; Stringaris & Goodman, 2009b) and at present, we cannot be certain whether the association between irritability symptoms and adolescent depressive symptoms is better accounted for by these other more well‐established pathways of anxiety/depressive symptoms and conduct problems.

With regard to early family risk factors, maternal depressive symptoms (pre‐ and postnatal) are robust and well researched risks for offspring depressive symptoms in adolescence (Pawlby, Hay, Sharp, Waters, & O'Keane, 2009; Pearson et al., 2013) and may act as a common antecedent of the three pathways outlined above (i.e. *Irritability Symptoms*;* Anxiety/Depressive Symptoms*; and *Conduct Problems;* Cents et al., 2013; Leis, Heron, Stuart, & Mendelson, 2014; Mars et al., 2012). Moreover, pre‐ and postnatal maternal depressive symptoms are associated with difficult (i.e. negative perceived mood, low perceived adaptability, and high perceived intensity/reactivity) early child temperament (Bruder‐Costello et al., 2007), which in turn, is associated with childhood anxiety/depressive symptoms and conduct problems (Barker & Maughan, 2009). Recently, Stringaris, Maughan, and Goodman (2010), reported that early temperamental dysregulation (emotionality and activity) predicted ODD diagnoses; however, the unique contribution of irritability symptoms was not examined. Yet, as irritability symptoms, anxiety/depressive symptoms, and conduct problems are highly comorbid, they may also share temperamental features.

Understanding whether there is a unique contribution of irritability symptoms toward adolescent depressive symptoms above anxiety/depressive symptoms and conduct problems may help refine risk to outcome associations and evidence‐based interventions. In addition, as little is known about the unique or shared temperamental antecedents of irritability symptoms, anxiety/depressive symptoms, and conduct problems, we explored associations of these child characteristics and toddler temperament (high perceived intensity, low perceived adaptability, and negative perceived mood). The three pathways were tested within an autoregressive cross‐lag model that allows us to test three possible equifinal pathways from the common family risk of maternal depressive symptoms toward adolescent depressive symptoms (e.g. Barker, Jaffee, Uher, & Maughan, 2011).

## Methods

### Sample

The Avon Longitudinal Study of Parents and Children (ALSPAC) was established to understand how genetic and environmental characteristics influence health and development in parents and children. All pregnant women residing in a defined area in the South West of England, with an expected date of delivery between 1st April 1991 and 31st December 1992, were eligible and 13,761 women (contributing 13,867 pregnancies) were recruited. These women have been followed over the last 19–22 years (Fraser et al., 2013). When compared with 1991 National Census Data, the ALSPAC sample was found to be similar to the UK population as a whole (Boyd et al., 2013). Ethical approval for the study was obtained from the ALSPAC Law and Ethics Committee and the Local Research Ethics Committees. More detailed information on ALSPAC is available from the website: http://www.bris.ac.uk/alspac/.

### Measures

Mothers completed questionnaires about their impressions of their own – and their children's – psychosocial wellbeing, at multiple time points during pregnancy, and their child's toddlerhood and childhood. The children reported their impressions of their own depressive symptoms at age 16.

Maternal depressive symptoms at 18 and 32 weeks prenatally and in the postnatal period at 8 weeks, 8 months, 21 months were assessed by asking mothers to complete the Edinburgh Postnatal Depression symptoms Scale Questionnaire (EPDS), a widely used 10‐item self‐report questionnaire that has been shown to be valid in and outside the postnatal period (Cox, Holden, & Sagovsky, 1987; Murray & Carothers, 1990). The EPDS is a 10 item self‐report questionnaire of symptoms experienced in the last 7 days and it has been used to identify pregnant women and mothers at risk of depressive symptoms. EPDS is a reliable measure of maternal depressive symptoms: prenatal (*α* = .78) and postnatal (*α* = .82).

Toddler temperament measures of Negative Perceived Mood, Low Perceived Adaptability and High Perceived Intensity at 24 months were used. Mothers completed each question using a 6‐point scale response, from ‘almost never’ to ‘almost always’ for each measure which are all Carey Infant Temperament sub‐scales (Carey & McDevitt, 1978). The ‘Negative perceived mood’ subscale is a measure of general tone of affect (i.e. positive or negative). Example items are, ‘he/she is fussy on waking up and going to sleep (frowns, cries)’, ‘he/she cries when left to play alone’. The ‘Low perceived adaptability’ subscale is a measure of responses to novel or altered situations. Examples of items are, ‘he/she resists changes in feeding schedule (1 hr or more) even after two tries, ‘he/she is still wary or frightened of strangers after 15 min’. The ‘High perceived intensity’ subscale is a measure of the level of energy with which an emotional response is made. Examples of items are, ‘he/she displays much feeling (vigorous laughing or crying) during nappy change or dressing, ‘he/she reacts strongly to strangers: laughing or crying’.

Irritability Symptoms at ages 8, 10, and 13 (mother and teacher reports) was derived from the Development and Well‐Being Assessment (DAWBA), a well‐validated measure, developed for the British Child Mental Health surveys (Meltzer, Gatward, Goodman, & Ford, 2000). In addition to generating binary (yes/no) diagnostic indicators, DAWBA algorithms have been developed to generate six‐level ordered‐categorical measures of the probability of disorder for each of the individual items underlying the diagnoses, ranging from <0.1% to >70% (Goodman, Heiervang, Collishaw, & Goodman, 2011). Evaluated in two large‐scale national samples, these DAWBA ‘bands’ functioned well as ordered‐categorical measures, showed dose–response associations with mental health service contacts, and showed very similar associations with potential risk factors as clinician‐rated diagnoses (Goodman et al., 2011).

The DAWBA asks 9 separate symptoms of ODD. Each question was introduced with the stem: ‘over the last 6 months, and as compared with other children the same age, has s/he often….’ followed by the specific clause. Following the lead of Stringaris and Goodman (2009a), and subsequently the DSM‐5 (American Psychiatric Association, 2013), irritability symptoms were defined by the following three symptoms: ‘has temper outbursts’, ‘has been touchy or easily annoyed’, and ‘has been angry or resentful’ where age 8–13 years (*α* = .71). Anxiety/Depressive symptoms at ages 7, 10, and 12 years were measured by mother reports on the Strengths and Difficulties Questionnaire, a widely used screening instrument with well‐established reliability and validity (Goodman, 1997, 2001; Van Widenfelt, Goedhart, Treffers, & Goodman, 2003) and anxiety/depressive symptoms has the following five items: ‘often complains of headaches, stomach aches or sickness’, ‘has many worries or often seems worried’, ‘often unhappy, depressed or tearful’,’is nervous or clingy in new situations, easily loses confidence’ and ‘has many fears, is easily scared’. Items were coded as a 3‐point scale (‘not true’, ‘somewhat true’, and ‘certainly true’) where age 7–12 years (*α* = .71). It should be noted that at age 7 the item ‘has many fears, is easily scared’ was not available in the dataset, but was included at ages 10 and 12.

Conduct Problems at ages 7, 10 and 12 years were measured by mother reports on the Strengths and Difficulties Questionnaire, a widely used screening instrument with well‐established reliability and validity (Goodman, 1997, 2001), with the following four items: ‘is generally obedient, usually does what adults request’ (reverse coded), ‘often fights with other children or bullies them’, ‘often lies or cheats, ‘steals from home, school, or elsewhere’. Items were coded as a 3‐ point scale (‘not true,’ ‘somewhat true,’ and ‘certainly true’) where age 7–12 years (*α* = .72). It should be noted that SDQ was found to be at least as efficient at detecting externalizing problems as the Child Behavior Checklist (CBCL; Goodman & Scott, 1999); and associates with ICD‐10 diagnoses of CD and ODD (Goodman, Renfrew, & Mullick, 2000). However, the temper outburst item that is typically the final measure for the SDQ for conduct problems was removed in this study to avoid item overlap between this item and the irritability temper tantrum/outburst item.

Depressive symptoms at age 16 were derived from the adolescent‐reported Mood and Feelings Questionnaire Short Form (SMFQ) (Messer et al., 1995). The SMFQ is a 13‐item self‐report questionnaire of symptoms experienced in the previous 2 weeks (that codes symptoms on a 3‐point scale: ‘true’, ‘sometimes true’, ‘not true’) with a range of 0–26 (*α* = .91). This scale has been found to have high reliability and validity, and the short form is made up of items that best discriminated depressed and nondepressed children in field trials using structured psychiatric interviews (Costello & Angold, 1988).

#### Attrition and missing data

Participants with data for depressive symptoms at 16 years were selected for the analysis (*n* = 3,963). In a multiple regression model, we tested the extent to which risk factors common to irritability symptoms, anxiety/depressive symptoms, and conduct problems (see Tremblay, 2010) associated with noninclusion in this study. Partner status (OR = 2.45; 95% CI = 2.00–2.99), low SES (OR = 1.68; 95% CI = 1.47–1.91), teen pregnancy (OR = 2.81; 95% CI = 2.53–3.12), and maternal education (OR = 2.57; 95% CI = 2.53–3.12). We note that inclusion of these variables in the analysis – in conjunction with missing data replacement by full‐information maximum likelihood – can help to minimize bias and maximize recoverability of ‘true’ scores (Little & Rubin, 2002).

### Analysis

**Figure 1:**
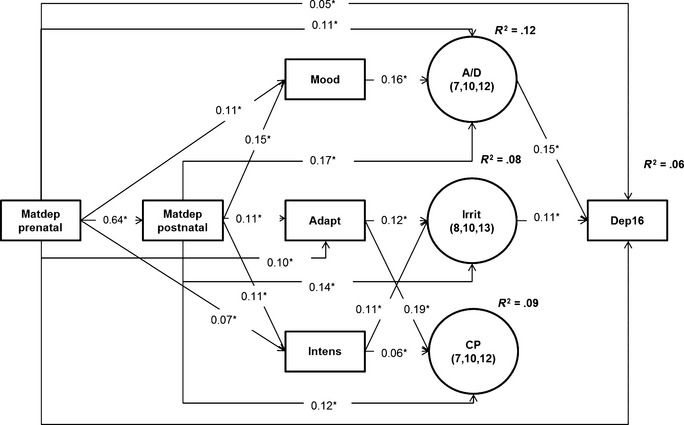
Multivariate Autoregressive cross‐lagged model of longitudinal relationships between maternal depressive symptoms, early toddler temperament, irritability symptoms, anxiety/depressive symptoms, and an outcome of adolescent depressive symptoms. Multivariate Autoregressive cross‐lagged model; * = *p *<* *.05; Matdep prenatal = Prenatal maternal depressive symptoms; Matdep postnatal = Postnatal maternal depressive symptoms; Mood = Negative perceived mood; Adapt = Low perceived adaptability; Intens = High perceived intensity; Irrit = Irritability symptoms at 8,10 and 13 years collapsed; A/D = Anxiety/depressive symptoms at 7,10 and 12 years collapsed; CP = Conduct problems at 7, 10 and 12 years collapsed; and Dep16 =  Adolescent depressive symptoms. In this model, we controlled for risk factors common to irritability, anxiety/depressive symptoms, and conduct problems and associated with noninclusion in this study. The resulting population effect sizes are interpreted using the Cohen (1988) guidelines: an effect of 0.10 is a small effect, an effect of 0.24 is a medium effect, and an effect of 0.37 is a large effect. Significant (*) results only are shown

Using an autoregressive cross‐lag model (ARCL), we tested three possible equifinal pathways from the common family risk of maternal depressive symptoms toward adolescent depressive symptoms (Figure 1). In this modeling approach, each variable in the model is regressed on all of the variables that precede it in time in order to examine developmental continuity and inter‐relationships across the three hypothesized pathways. The *Irritability Symptoms* pathway predicts associations among pre‐and postnatal maternal depressive symptoms, temperament, irritability symptoms and adolescent depressive symptoms; the *Anxiety/Depressive Symptoms* pathway predicts associations among pre‐ and postnatal maternal depressive symptoms, temperament, anxiety/depressive symptoms and adolescent depressive symptoms; the *Conduct Problems* pathway predicts associations between pre‐ and postnatal maternal depressive symptoms, temperament, conduct problems and adolescent depressive symptoms.

## Results

### Descriptive statistics

All study variables were significantly positively correlated (Table 1). For example, negative perceived mood, low perceived adaptability and high perceived intensity were all highly correlated. Moreover, childhood irritability symptoms, anxiety/depressive symptoms and conduct problems were highly correlated. Adolescent depressive symptoms were significantly associated with maternal depressive symptoms, toddler temperaments and child irritability symptoms, anxiety/depressive symptoms and conduct problems. We note that the highest correlation was between pre‐ and postnatal maternal depressive symptoms (*r = *.64), which indicated that these measures shared 41% common variance (i.e. 0.64*0.64 = 0.41).

**Table 1:** Bivariate correlations of the study variables

	1.	2.	3.	4.	5.	6.	7.	8.	9.
Prenatal Matdep	–	–	–	–	–	–	–	–	–
Postnatal Matdep	0.64^a^	–	–	–	–	–	–	–	–
Mood	0.21^a^	0.22^a^	–	–	–	–	–	–	–
Adapt	0.17^a^	0.18^a^	0.58^a^	–	–	–	–	–	–
Intens	0.14^a^	0.15^a^	0.37^a^	0.40^a^	–	–	–	–	–
A/D	0.05^a^	0.06^a^	0.24^a^	0.17^a^	0.12^a^	–	–	–	–
Irrit	0.05^a^	0.05^a^	0.16^a^	0.20^a^	0.18^a^	0.49^a^	–	–	–
CP	0.05^a^	0.06^a^	0.18^a^	0.25^a^	0.17^a^	0.35^a^	0.61^a^	–	–
Dep16	0.09^a^	0.08^a^	0.06^a^	0.05^a^	0.04^a^	0.19^a^	0.16^a^	0.08^a^	–

a *p *<* *.01.

Matdep prenatal = Prenatal maternal depressive symptoms; Matdep postnatal = Postnatal maternal depressive symptoms; Mood = Negative perceived mood; Adapt = Low perceived adaptability; Intens = High perceived intensity; Irrit = Irritability symptoms; A/D = anxiety/depressive symptoms; CP= Conduct problems; Dep16 = Adolescent depressive symptoms.

### Examining three pathways to adolescent depressive symptoms

The ARCL model showed acceptable fit on three indices (*χ* ^2^ (121) = 471.978, *p* < .001; CFI: 0.964; TLI: 0.953; RMSEA: 0.027). *R* ^2^ values (the proportion of variance explained based on each variable's predictors) are reported for adolescent depressive symptoms (*R* ^2^ = .06); irritability (*R* ^2^ = .08); anxiety/depressive symptoms (*R* ^2^ = .12) and conduct problems (*R* ^2^ = .09).

Figure 1 shows the significant path coefficients in the ARCL model. To begin with, we note that prenatal maternal depressive symptoms were associated with postnatal maternal depressive symptoms (*β* = .64). In addition, we highlight four main results. First, for the *Irritability Symptoms* pathway: postnatal maternal depressive symptoms was associated with low perceived adaptability (*β* = .11) and high perceived intensity (*β* = .11) in toddlerhood, which were associated with irritability symptoms at 8–13 years (*β* = .12; *β* = .11; respectively), which, in turn, was associated with adolescent depressive symptoms (*β* = .11). Second, for the *Anxiety/Depressive Symptoms* pathway: postnatal maternal depressive symptoms were associated with toddler negative perceived mood (*β* = .15), which was associated with anxiety/depressive symptoms at 7–12 years (*β* = .16), which, in turn, was associated with adolescent depressive symptoms (*β* = .15). Third, for the *Conduct Problems* pathway: postnatal maternal depressive symptoms were associated with low perceived adaptability (*β* = .11) and high perceived intensity (*β* = .11), which were associated with conduct problems at 7–12 years (*β* = .19; *β* = .06; respectively). Conduct problems at 7–12 years did not predict adolescent depressive symptoms, above and beyond the other variables in the ARCL model.

Fourth, in addition, direct associations were found between prenatal depressive symptoms and anxiety/depressive symptoms at 7–12 years and depressive symptoms at 16 years (*β* = .11; *β* = .05, respectively). Direct associations were also found between postnatal depressive symptoms and irritability (*β* = .14); anxiety/depressive symptoms (*β* = .17); and conduct problems (*β* = .12).

## Discussion

The present epidemiological study examined three distinct pathways linking a common antecedent – maternal depressive symptoms – to a shared equifinal outcome of adolescent depressive symptoms. The overall findings provide evidence for two distinct co‐occurring pathways from maternal to adolescent depressive symptoms: an *Irritability Symptoms* pathway and an *Anxiety/Depressive Symptoms* pathway; however a third *Conduct Problems* pathway was not found.

With regard to irritability symptoms (i.e. temper outbursts, being easily annoyed and angry or resentful), unlike previous studies (e.g. Stringaris et al., 2010; Whelan et al., 2013), this study examined irritability symptoms while controlling for co‐occurring anxiety/depressive symptoms and conduct problems. Study findings delineate the specific contribution of childhood irritability symptoms to adolescent depressive symptoms, alongside its earlier temperamental features. More specifically, we found a pathway from pre‐ and postnatal maternal depressive symptoms, to temperamental low perceived adaptability and high perceived intensity in toddlerhood, to childhood irritability symptoms, and ultimately to adolescent depressive symptoms. With regard to temperamental low perceived adaptability, studies have found that irritable children perform poorly in tasks of cognitive flexibility thereby demonstrating deficits (Adleman et al., 2011; Leibenluft, 2011) – that is, low perceived adaptability. It may therefore be that irritable children display early temperamental signs of cognitive and behavioral inflexibility (i.e. low perceived adaptability) which manifest as prodromal signs of irritability symptoms, which, if recognized early, may offer a treatment target. In addition, the association that we found between toddler high perceived intensity and childhood irritability symptoms may be explained by the fact that biological systems relevant to the regulation of arousal are functionally immature during pregnancy and birth and mature gradually during the toddler years (Glover, 2011); postnatal maternal depressive symptoms may impact adversely upon the development of these systems. During infancy, the child is dependent on parenting (Jaffee, 2007) to support the achievement of developmental milestones such as cognitive maturation and early social and emotional competence (Shonkoff, Boyce, & McEwen, 2009). However, the presence of depressive symptoms may compromise a mother's ability to provide the sensitive care needed to foster the development of the toddler's self‐regulatory capabilities (Barker, 2013; Feldman et al., 2009; Goodman & Gotlib, 1999).

Childhood anxiety/depressive symptoms – worrying, being unhappy and tearful and fearful –were found to uniquely contribute to adolescent depressive symptoms above and beyond childhood irritability symptoms and conduct problems, thereby providing a second pathway to depressive symptoms at 16 years. This second pathway linked pre‐ and postnatal maternal depressive symptoms with toddler temperamental negative perceived mood, childhood anxiety/depressive symptoms, and adolescent depressive symptoms. The associations between pre‐ and postnatal maternal depressive symptoms and child anxiety/depressive symptoms may be explained by two distinct but related processes. With regard to prenatal depressive symptoms, our findings appear congruent with research suggesting that depressive symptoms can lead to an intra‐uterine environment not conducive to healthy fetal development (Weinstock, 2008), thereby increasing risk for abnormal child development, including –but not specific to – childhood anxiety/depressive symptoms (Barker et al., 2011; Glover, 2011). Additionally, as noted above with respect to irritability symptoms, postnatal depressive symptoms may negatively alter a mother's ability to provide attentive and sensitive care needed to foster the development of the toddler's self‐regulatory capabilities (Barker, 2013; Feldman et al., 2009; Goodman & Gotlib, 1999). This in turn could increase the risk of a toddler developing temperamental negative perceived mood and childhood anxiety/depressive symptoms, ultimately increasing the risk for the onset of adolescent depressive symptoms. Future research may want to examine more closely how specific symptoms of postnatal maternal depressive symptoms (e.g. as measured by the EPDS: anxious or worried vs. low laughter, humor) may align more as a risk for anxiety/depressive symptoms or irritability.

Third, conduct problems were not found to associate with adolescent depressive symptoms when childhood irritability symptoms and anxiety/depressive symptoms were controlled. In this study, at the bivariate level, the correlation between conduct problems and depressive symptoms was significant, albeit half the magnitude of the association between irritability symptoms and adolescent depressive symptoms. However, in the autoregressive cross‐lagged model, this association became nonsignificant. Possibly, conduct problems associate with adolescent depressive symptoms (e.g. Barker et al., 2010; Lahey et al., 2002) via irritability symptoms. Indeed, a recent study (Stringaris et al., 2014) highlighted that irritability symptoms shared genetic associations with childhood depressive symptoms and conduct problem symptoms.

Strengths of this study include large sample size, longitudinal focus, and inclusion of cross‐informant predictions (i.e. mother and teacher reports of risks, child reports of adolescent depressive symptoms). However, the present results should be interpreted in light of a number of limitations. First, this study, as with previous studies, is correlational and not causative. Second, it should be noted that we relied on self‐reports of mothers on a range of study variables including their own depressive symptoms and the child temperaments and irritability, anxiety/depressive symptoms and conduct problems. An important limitation is that the study almost exclusively (exception being adolescent depressive symptoms) relied on mothers’ impressions of their own – and their child's – psychosocial wellbeing (e.g. hence ‘perceived’ temperament) Indeed, although studies suggest that depressed mothers can be as accurate as other informants about their children's behavior (Richters, 1992), ALSPAC does not have the capability of confirming or disconfirming potential bias associated with maternal depressive symptoms by comparing mother reports of their children to independent, validated criterion raters. Third, we do not have information on whether mothers in this study received treatment for depression. As treatment induced reduction in maternal depressive symptoms associates in improved adjustment in offspring (Pilowsky et al., 2008; Weissman et al., 2006), the present results may underestimate the effect of maternal depression on child wellbeing. Fourth, younger and more socially disadvantaged mothers were more likely to be lost to follow‐up. As these predictors of attrition also predict child psychopathology, our sample is likely to under‐represent the most severely affected children. Of note, an ALSPAC cohort study (Wolke et al., 2009) has shown that attrition affects the prevalence of DSM‐IV disruptive behavior disorders (which includes ODD), however, associations between risks and outcomes remained present, although conservative of the likely true effects. Fifth, although the ALSPAC sample represents a broad spectrum of SES backgrounds, it includes relatively low rates of ethnic minorities. The present results will need replication with more ethnically diverse and high risk samples. Sixth, this study did not test for indirect effects between study variables and as such future studies may wish to examine indirect effects. If indirect effects were found they would suggest that successful intervention on maternal depressive symptoms could lead to lower adolescent depressive symptoms through higher perceived toddler temperamental adaptability and lower perceived intensity, and lower irritability symptoms.

In conclusion, we found that irritability symptoms contributed independently to adolescent depressive symptoms when co‐occurring anxiety/depressive symptoms and conduct problems were controlled. Moreover, common risk factors of pre‐ and postnatal maternal depressive symptoms were associated with two equifinal pathways to adolescent depressive symptoms, based on temperamental features and child characteristics. First, we found, an *Irritability Symptoms* pathway linked with toddler temperamental low perceived adaptability and high perceived intensity. Second, we also found an *Anxiety/Depressive Symptoms* pathway linked with toddler temperamental negative perceived mood. Thus, this study supports the existence of distinct developmental pathways to adolescent depressive symptoms while pinpointing important targets and windows of opportunity for prevention. We suggest that interventions addressing childhood irritability symptoms, as well as maternal depressive symptoms, toddler temperamental low perceived adaptability and high perceived intensity; and those that target childhood anxiety/depressive symptoms alongside toddler temperamental negative perceived mood may be the most efficient manner to prevent the onset of adolescent depressive symptoms.

**Key points**

- Studies suggest that the ODD subdimension of irritability prospectively associates with adolescent and young adult depressive symptoms
- This study supports the existence of a distinct Irritability Symptoms developmental pathway to adolescent depressive symptoms above and beyond co‐occurring anxiety/depressive symptoms and conduct problems.
- Common risk factors – maternal depressive symptoms were associated with two equifinal pathways to adolescent depressive symptoms, based on temperamental features and child characteristics. An Irritability Symptoms pathway linked with toddler temperamental low adaptability and high intensity; and an Anxiety/Depressive Symptoms pathway linked with toddler temperamental negative mood.
- We suggest that interventions addressing childhood irritability symptoms, as well as childhood anxiety/depressive symptoms alongside toddler temperamental negative mood may be the most efficient manner to prevent the onset of adolescent depressive symptoms.
